# Chemical composition modulates the adverse effects of particles on the mucociliary epithelium

**DOI:** 10.6061/clinics/2015(10)09

**Published:** 2015-10

**Authors:** Regiani Carvalho-Oliveira, Ruy Camargo Pires-Neto, José Oscar Viega Bustillos, Mariangela Macchione, Marisa Dolhnikoff, Paulo H. Nascimento Saldiva, Maria Lúcia Bueno Garcia

**Affiliations:** I Faculdade de Medicina da Universidade de São Paulo, Departamento de Patologia, Laboratório Experimental de Poluição Atmosférica, São Paulo, SP, Brazil; II Instituto de Energia e Pesquisa Nuclear, São Paulo, SP, Brazil

**Keywords:** Mucociliary Epithelium, Ciliary Beat Frequency, Mucociliary Transport, PM_2.5_, Total Suspended Particles

## Abstract

**OBJECTIVE:**

We compared the adverse effects of two types of real ambient particles; i.e., total suspended particles from an electrostatic precipitator of a steel mill and fine air particles from an urban ambient particulate matter of 2.5 µm, on mucociliary clearance.

**METHOD:**

Mucociliary function was quantified by mucociliary transport, ciliary beating frequency and the amount of acid and neutral mucous in epithelial cells through morphometry of frog palate preparations. The palates were immersed in one of the following solutions: total suspended particles (0.1 mg/mL), particulate matter 2.5 µm 0.1 mg/mL (PM0.1) or 3.0 mg/mL (PM3.0) and amphibian Ringer's solution (control). Particle chemical compositions were determined by X-ray fluorescence and gas chromatography/mass spectrometry.

**RESULTS:**

Exposure to total suspended particles and PM3.0 decreased mucociliary transport. Ciliary beating frequency was diminished by total suspended particles at all times during exposure, while particulate matter of 2.5 µm did not elicit changes. Particulate matter of 2.5 µm reduced epithelial mucous and epithelium thickness, while total suspended particles behaved similarly to the control group. Total suspended particles exhibited a predominance of Fe and no organic compounds, while the particulate matter 2.5 µm contained predominant amounts of S, Fe, Si and, to a lesser extent, Cu, Ni, V, Zn and organic compounds.

**CONCLUSION:**

Our results showed that different compositions of particles induced different airway epithelial responses, emphasizing that knowledge of their individual characteristics may help to establish policies aimed at controlling air pollution.

## INTRODUCTION

It is widely accepted that atmospheric particulate matter (PM) exposure is associated with adverse health effects, especially in the airway epithelium 1-2. Air pollutants affect nasal and lung functions, which trigger clinical symptoms 3-4 and induce alterations in mucociliary apparatus properties and defenses, predisposing to respiratory infections 5.

The mucociliary apparatus represents the lung's first line of defense against inhaled noxious agents by removing particles and chemical species from the airways through continuous transportation of airway mucus to the oropharynx, using the mechanical input provided by the coordinated beating of the cilia. In this context, the mucociliary apparatus represents a critical point of interaction between the defenses of the organism and inhaled toxicants and such contact modulates the development of adverse respiratory effects promoted by air pollution.

Real ambient particles are toxic even in healthy people and within standard levels according to international guidelines, as they induce modest but continuous alterations of the respiratory system 6. Studies on the mucociliary apparatus in humans exposed to real ambient fine particles are usually epidemiological studies, which do not search for causality. However, controlled experimental studies designed to detect the mechanistic pathways of hazard effects caused by ambient pollutants are frequently based on artificially manipulated or concentrated ambient particle exposure to obtain significant data. However, artificially concentrated PM does not reflect real-world pollutant exposure. Consequently, few experimental studies have focused on different chemical components of real ambient PM and their physiopathologic effects on the mucociliary apparatus.

Airborne PM consists of a complex mixture of particles of varying sizes and physicochemical compositions. The chemistry of ambient particles is dependent on several factors, including the sources of the particulate pollution in the local environment 7. An understanding of the pathogenesis of adverse effects of ambient PM on the respiratory epithelium is hindered by the heterogeneity of sources, and, consequently, variations in size, physical characteristics and chemical composition 6. Biological effects are even more pronounced for particles smaller than 2.5 μm (PM_2.5_), commonly referred to in the literature as the fine fraction of particulate matter 7. The adverse effects of PM_2.5_ have been associated with its weight, mass, particle number and size in special ultrafine particles, along with its surface area, surface chemistry and chemical specification, such as metals 8-9 or polyaromatic hydrocarbons (PAH) 10-11, which depend on the source of the particulate matter. Because of the multiple pathways of damage exhibited by the aforementioned particle characteristics, some of the physiopathological responses to PM_2.5_ remain unclear.

The Health and Sustainability Institute Report 12 evaluated São Paulo in Brazil from 2006 to 2011. The PM_2.5_ levels were collected from a large metropolis; i.e., São Paulo city, which predominantly has pollutants from vehicular sources followed by industrial sources. During the analyzed period, there were 7,932 deaths/year due to air pollution in the state of São Paulo and 4,655 deaths/year in São Paulo city, corresponding to 99,084 deaths/5 years in the state, which is similar to an entire city of 100,000 inhabitants dying over 5 years. Air pollution caused more deaths than traffic and infectious diseases, such as tuberculosis and acquired immune deficiency syndrome (AIDS). Respiratory morbidities and deaths were the most frequent respiratory diseases in adults associated with air pollutants (38%), followed by cardiovascular events (33%). The disability-adjusted life-year (DALY) and economic wasting indexes presented correlations with air pollutant concentration oscillations. This study clearly stated the impact of air pollutant levels on health in São Paulo and that consideration of air pollutants' impact on health is worthwhile. However, it did not analyze the air pollutants' composition, their source and their mechanistic pathway of injury 12.

Therefore, the present study aimed to compare adverse effects of PM from different sources, specified as total suspended particles (TSP) collected from an electrostatic precipitator of the steel-sintering plant from an industrial area in São Paulo state and fine atmospheric particles from real urban ambient PM_2.5_ from São Paulo city, on the mucociliary apparatus.

## MATERIALS AND METHODS

### Experimental Design

**Figure 1:**
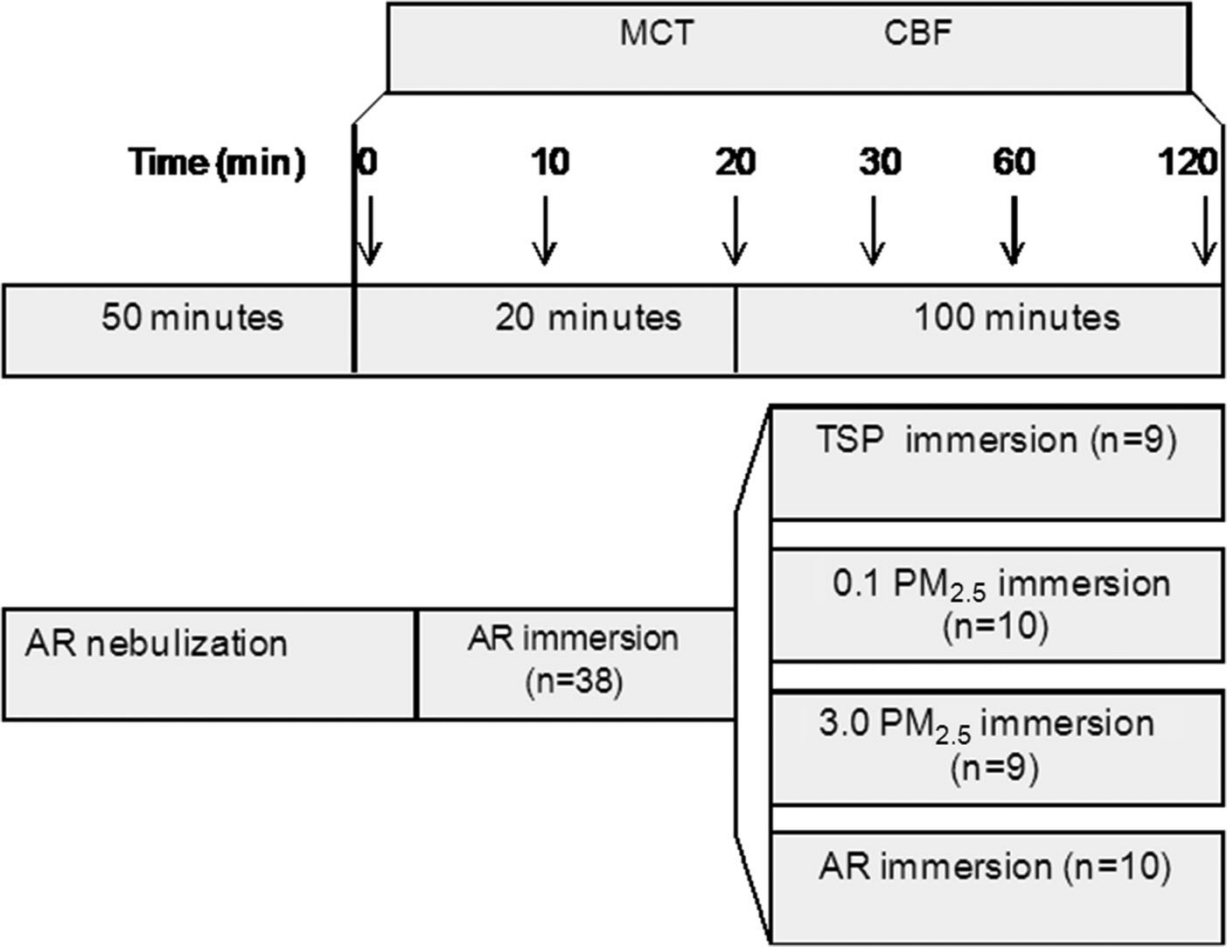
Schematic representation of the experimental protocol. Particle immersions were performed with TSP (0.1 mg/mL) or PM_2.5_ (0.1 and 3.0 mg/mL), and the negative control group was immersed in amphibian ringer (AR) solution. Arrows represent the time of mucociliary transport and ciliary beating frequency measurements. TSP, total suspension particles collected from an electrostatic precipitator of a steel plant; 0.1 PM2.5, urban particulate matter 2.5 μm in aerodynamic diameter at a 0.1 mg/mL concentration; 3.0 PM2.5, urban particulate matter 2.5 μm in aerodynamic diameter at a 3.0 mg/mL concentration.

Thirty-eight frog palates were submitted to nebulization in amphibian Ringer's (AR) solution for 50 minutes, after which the basal measurements (time 0) for mucociliary transport (MCT) and ciliary beating frequency (CBF) were made. The frog palates were immersed in AR solution for 20 minutes to evaluate the effects in an aqueous medium. After this period, these palates were divided into 4 groups and immersed in different solutions as follows: AR solution containing 0.1 mg/mL of TSP (TSP group, n=9); AR solution containing 0.1 mg/mL of PM_2.5_ (PM0.1 group, n=10); AR solution containing 3.0 mg/mL of PM_2.5_ (PM3.0 group, n=9); and AR, the negative control group (n=10). The MCT and the CBF were measured at 10, 20, 30, 60 and 120 minutes of immersion. After the exposure time (120 minutes), fragments of the epithelia of all frog palates were taken and fixed in 10% buffered formalin solution for posterior morphometric analyses. A schematic representation of the experimental design is shown in Figure 1.

### Particle Sampling

TSP was collected from an electrostatic precipitator of a steel-sintering plant. For experimental testing on frog palates, TSP were suspended in AR solution (Ringer's solution mixed with distilled water, 1:1 in volume) with 50 minutes of ultra-sonication using a Sonifier model T14 (L&R Manufacturing Company, Kearny, New Jersey, USA). PM_2.5_ was sampled on June 17, July 21, July 23, July 24 and August 21 in 2003. For this purpose, we employed a high-volume sampler (Energetica, Brazil) coupled with an inlet (Tisch Environmental Inc., USA) that allowed the separation of particles below 2.5 μm at a flow rate of 1.1 cubic meters per minute over 24 hours. Our particle sampler was located on the rooftop of our Medical School (approximately 15 meters above the ground), which is situated on a heavy-traffic crossroad in downtown São Paulo, Brazil. Particles were collected in glass fiber filters (Energetica, Brazil), which were left to dry for 24 hours at 50 degrees Celsius before and after particle collection for weighing. After drying, the filters were kept in a refrigerator at 4°C for future analyses. For the evaluation of PM_2.5_ toxicity, particles first had to be extracted from filters and were then ultrasonicated in AR using the same apparatus described above. This step of the experiment lasted 8 hours. The efficiency of extraction was determined by weighing the filters before and after extraction. The weight of the filters was determined after drying them for 24 hours at 50°C.

### Trace Elements Determination

For determination of the elemental composition, the PM_2.5_ samples were collected in 5 polycarbonate filters (Isopore™ Membrane Filters Polycarbonate, 0.8 μm, 37 mm, Millipore, Billerica, MA, USA) using Harvard Impactors (Air Diagnostics, Harrison, ME) operating at 10 L/min for 24 hours 13 in the same period and location of the samples used in the toxicological study. For determination of the TSP elemental composition, 0.5 g of the TSP sample was transformed into a 20-mm-diameter pellet by applying a pressure of 636.62 MPa for 60 s with a mechanical press (TECLAGO, Model PCA 40). Thus, three pellets were made, which were analyzed twice using the same methodology. Metallic and non-metallic contents were determined using energy-dispersive X-ray fluorescence spectroscopy (EDXRF; EDX 700-HS, Shimadzu Corporation Analytical Instruments Division, Kyoto, Japan). This instrument used a low-power Rh-target tube, a voltage range of 5 kV to 50 kV and a current of 1 to 1,000 μA. The characteristic X-ray radiation was detected with an Si (Li) detector. X-ray fluorescence emission spectra of the high-energy (Ti-U) and low-energy (Na-Sc) elements were collected for 240 and 400 s, respectively, from a sample surface area of 10 mm under a vacuum atmosphere 14. The measured sample intensities were converted to element concentrations (ng cm^-2^) with calibration of the NIST 2783 (Air Particulate on Filter Media) standard reference materials (National Institute of Standards, Gaithersburg, MD, USA). Finally, carbon was used for mass balance. The precision and accuracy of this methodology were verified using the standard reference material (NIST 2783; Appendix 1).

### Organic Compound Comparative Determination

Qualitative determination of organic compounds was conducted, comparing samples between TSP and urban PM_2.5_. The analysis was performed in five polycarbonate filters and in 1 g of TSP, after extraction with pentane, with gas chromatography/mass spectrometry (GC/MS QP5000, Shimadzu Corporation Analytical Instruments Division, Kyoto, Japan). The pentane solvent was part of the analysis, but its contribution was discounted from the results.

### Frog Palate Preparation

Mature *Rana catesbiana* frogs weighing approximately 100 g were obtained from the vivarium in the city of Mogi das Cruzes. At the vivarium, all frogs received the same care and were in the same environmental conditions. In our laboratory, the animals received a balanced diet and water *ad libitum*, according to routine veterinary procedures, until their sacrifice. All animals received humane care in compliance with the Helsinki convention for the use and care of animals. Using hypothermia as an anesthesia, each frog was rapidly decapitated, the jaw was disarticulated and the palate was removed through a cut made from the junction of the posterior pharynx and esophagus out to the skin of the back. The excised palate was placed on a piece of gauze saturated with AR and then placed on a dish loosely covered with plastic wrap and allowed to rest in a refrigerator at 4°C for 2 days. On the third day, mucus samples from the posterior edge of the palate were collected with a needle and immediately immersed in mineral oil to prevent dehydration. All of the experiments were performed on the third day. Under these experimental conditions, the ciliary activity was maintained 15.

### *Ex situ* Mucociliary Transport

MCT by ciliary beating was evaluated using the *in vitro* frog palate preparation. Determination of MCT was made by measuring the rate of displacement of autologous mucus samples, placed on the epithelial surface of the frog palate, using a stereoscopic microscope equipped with a reticulated eyepiece. MCT was calculated by dividing the distance traveled (6 mm) by the elapsed time (s). At least five measurements were made for each sample and time of the study. The mucus samples were rinsed with petroleum ether to remove the oil prior to their placement on the surface of the palate. The experiments were performed at room temperature (20°C). During the measurements of the MCT, the frog palate was maintained inside an acrylic chamber with a micro-environment of 100% humidity, provided through ultrasonic nebulization of AR 15.

### Ciliary Beat Frequency

CBF was measured through a modification of the videoscopic technique. It consisted of focusing on a group of cilia through an optical microscope (10X objective, 10X ocular) connected to a video camera (Sony, mod. 3CCD Iris), with the resulting image sent to a monitor (Sony Trinitron). A stroboscopic light (Machine Vision Strobe, mod. 5000, USA) was placed in front of the ciliary epithelium and the light emitted flashes (0 Hz to 30 Hz). The incident light illuminating the ciliated epithelium was reflected from the cilia packed together and from the thin layer of mucus covering the cilia. This reflection was cyclic because its direction changed according to movements of the underlying cilia. Through manual control, it was possible to define the ciliary frequency activity when it was the same as the flash frequency when the observer could not distinguish the ciliary beat clearly 15.

### Morphometric Analyses

After the exposure time, fragments of the palate epithelia of all palates were taken, fixed in 10% buffered formalin solution and processed according to routine histological procedures for paraffin embedding and cutting. Transversally oriented 5-μm-thick slices were taken and stained with the combination of Schiff's periodic acid (PAS) and Alcian Blue (AB) at a pH 2.5. With this technique, neutral and acid glycoproteins were stained in red (PAS) and blue (AB), respectively. Quantitative morphologic evaluation of the frog palate epithelium was made by means of a point-counting technique. A graticule of 100 points attached to the eyepiece of an optical microscope was used and 1,000x magnification was selected. For each slide, 1,000 points were counted in 10 randomly selected microscopic fields. This number was large enough to keep the coefficient of error (SE/mean) under 10%. The volume proportion of intraepithelial acid mucus (AM) and intraepithelial neutral mucus (NM) was assessed by counting the number of points hitting each type of mucus, empty spaces and the non-secretor area of the epithelium in each field. We then calculated the number of points corresponding to the total area of the epithelium in each field. The volume proportion of mucus substance or empty cells was calculated using the ratio between the number of points hitting the mucosubstance (acidic or neutral) or empty cells and the total number of points hitting the epithelium. The volume proportion was expressed as a percentage.

The volume proportion of total mucus (TM) was calculated as AM + NM. Frog palate epithelium thickness (ET) was assessed by dividing the total number of points hitting the epithelium by the corresponding basal membrane length.

### Statistical Analysis

Statistical analyses for MCT and CBF were performed using general linear models for repeated measurements, containing categorical indicators of treatment and terms for time and treatment interaction. Post hoc tests were also employed (least difference in significance) for multiple comparisons. For morphometric analyses, the one-way analysis of variance (ANOVA) was performed. Post hoc tests were also employed (least difference in significance) for multiple comparisons. The SPSS v17.0 computer package was used for statistical analyses. The significance level was set at 5%.

### Ethics

This study was approved by the Ethics Committee of the School of Medicine, University of São Paulo.

## RESULTS

The results of TSP and urban PM_2.5_ of trace elements determination by X-ray fluorescence spectrometry are shown in Table 1. As expected, TSP and PM_2.5_ expressed different compositions. TSP exhibited a predominance of iron, which represented 64% of the total mass of the analyzed elements, while PM_2.5_ presented a heterogeneous composition, with the predominance of sulfur, sodium, calcium iron and silicon and, to a lesser extent, aluminum, zinc, copper, vanadium, nickel, titanium and lead. No organic compounds were detected through gas chromatography/mass spectrometry in the TSP sample, while p-xylene, toluene and ethyl benzene were identified in the PM_2.5_ filter samples.

**Figure 2:**
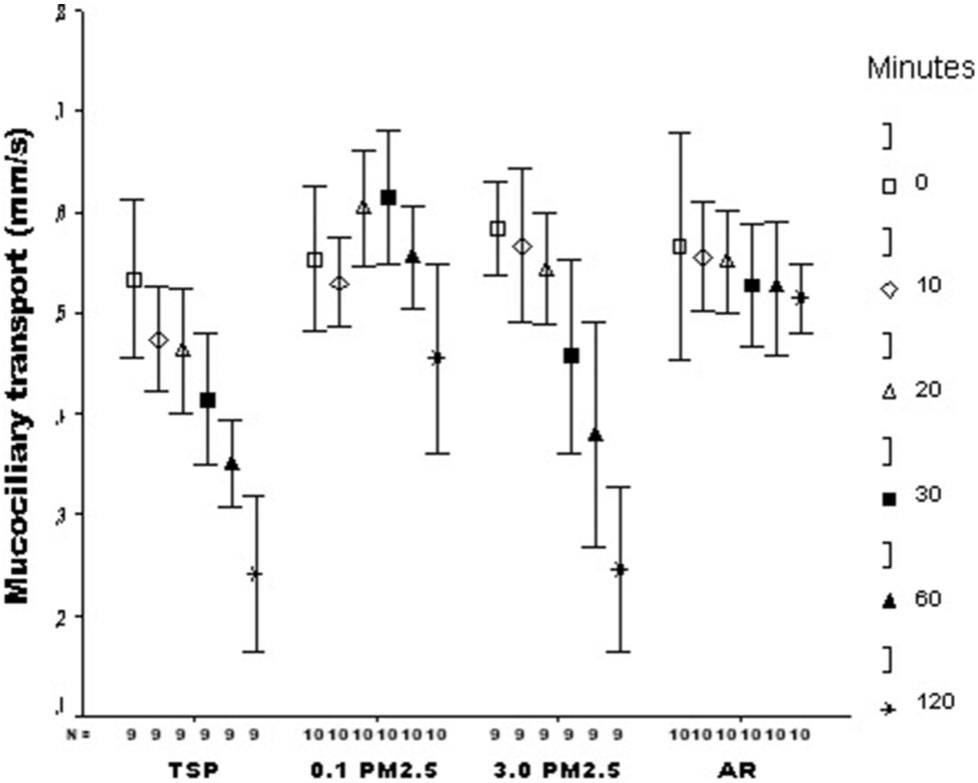
Mucociliary transport of all experimental groups at 0, 10, 20, 30, 60 and 120 minutes. Values are expressed as means ± SE. TSP and 3.0 mg/mL of urban particulate matter presented significantly lower values than AR and 0.1 mg/mL of urban particulate matter at all times of exposure (*p*<0.001). TSP, total suspension particles collected from an electrostatic precipitator of a steel plant; 0.1 PM2.5, urban particulate matter 2.5 μm in aerodynamic diameter at a 0.1 mg/mL concentration; 3.0 PM2.5, urban particulate matter 2.5 μm in aerodynamic diameter at a 3.0 mg/mL concentration.

Figure 2 presents the MCT results for all experimental times. Exposure to TSP and to 3.0 mg/mL of PM_2.5_ induced decreases in MCT (*p*<0.001) when compared to the other groups. The time of exposure exhibited interactions with the treatments employed, which started after 10 min of exposure (Time 30) and lasted up to 120 minutes (*p*<0.001). The effects of AR on MCT were similar to 0.1 mg/mL of PM_2.5_.

**Figure 3:**
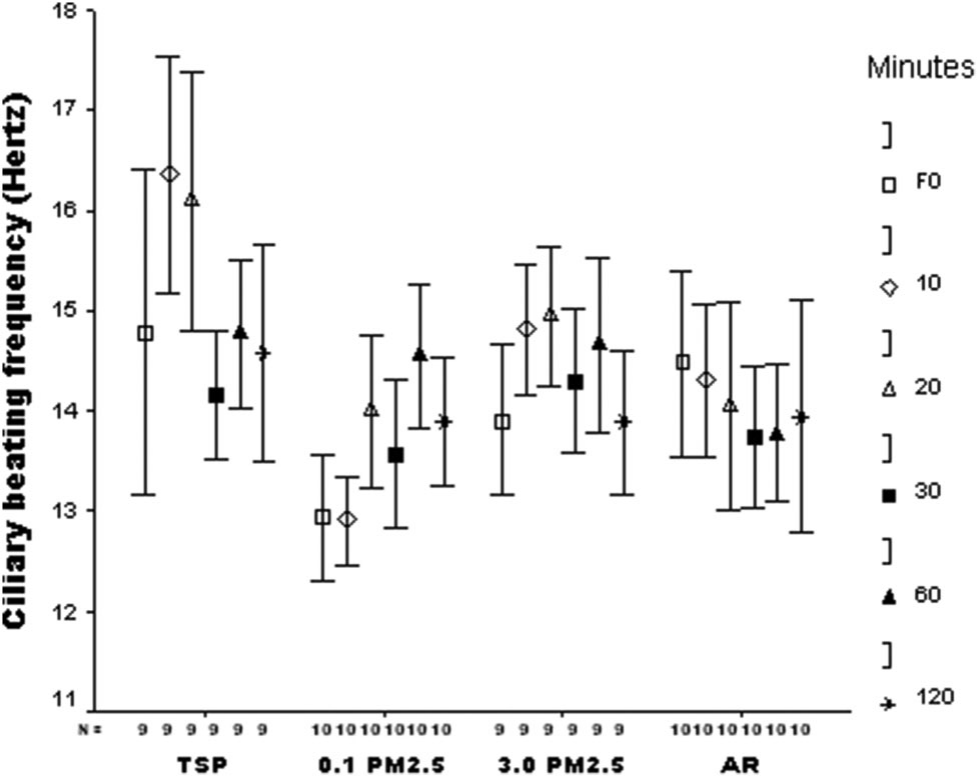
Ciliary beating frequencies of all experimental groups at 0, 10, 20, 30, 60 and 120 minutes. Values are expressed as means ± SE. TSP and 3.0 mg/mL of urban particulate matter presented significantly lower values than AR and 0.1 mg/mL of urban particulate matter at all times of exposure (p<0.001). TSP, total suspension particles collected from an electrostatic precipitator of a steel plant; 0.1 PM2.5, urban particulate matter 2.5 μm in aerodynamic diameter at a 0.1 mg/mL concentration; 3.0 PM2.5, urban particulate matter 2.5 μm in aerodynamic diameter at a 3.0 mg/mL concentration.

Figure 3 presents the results of CBF measurements. Exposure to TSP decreased CBF at all experimental times (*p*=0.011), while the PM and AR groups did not show any changes.

**Figure 4:**
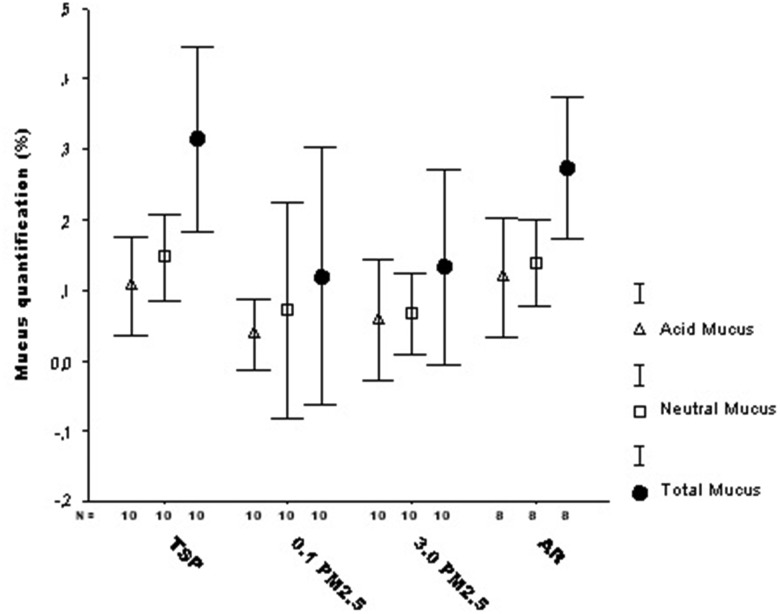
Mucus quantification in all groups, expressed as acid mucus (AM), neutral mucus (NM), and total mucus (TM). Values are expressed as means ± SE. Urban particulate matter in both concentrations showed decreases of the AM, NM and TM values when compared with the TSP and AR groups (*p*<0.001). TSP, total suspension particles collected from an electrostatic precipitator of a steel plant; 0.1 PM2.5, urban particulate matter 2.5 μm in aerodynamic diameter at a 0.1 mg/mL concentration; 3.0 PM2.5, urban particulate matter 2.5 μm in aerodynamic diameter at a 3.0 mg/mL concentration.

**Figure 5:**
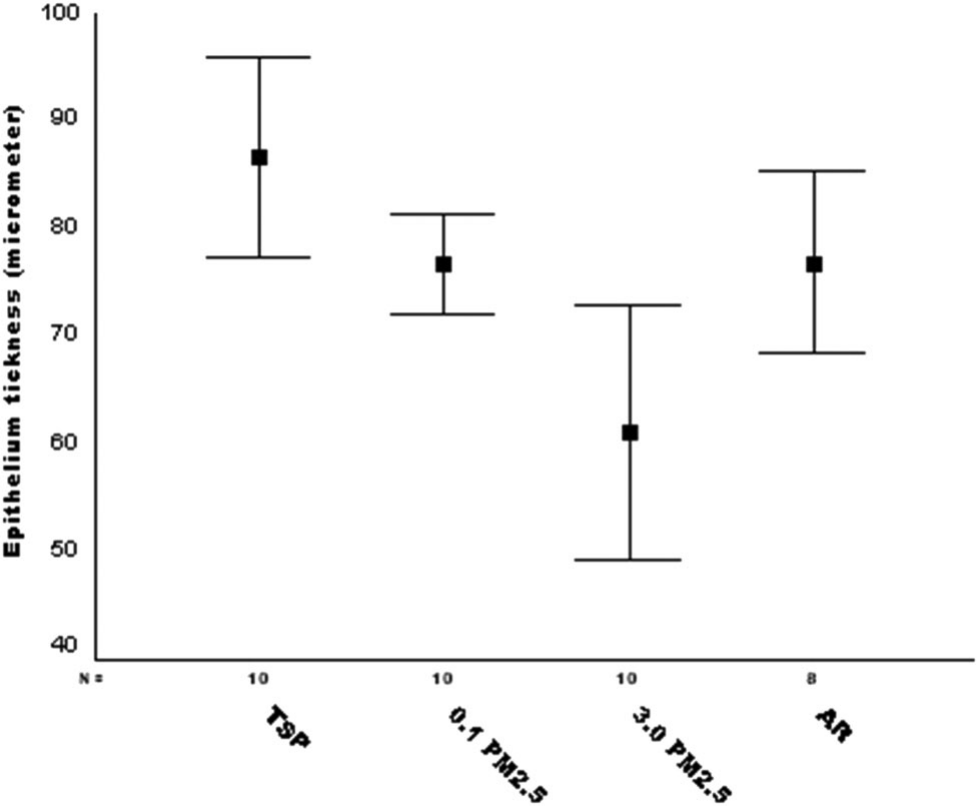
Epithelium thicknesses in all groups expressed as TSP, PM3, PM0.1 and AR. Values are expressed as means ± SE. Urban particulate matter (PM_2.5_) at a higher exposure concentration induced a decrease in the epithelium thickness (*p*=0.01), while TSP and AR did not induce any change. TSP, total suspension particles collected from an electrostatic precipitator of a steel plant; 0.1 PM2.5, urban particulate matter 2.5 μm in aerodynamic diameter at a 0.1 mg/mL concentration; 3.0 PM2.5, urban particulate matter 2.5 μm in aerodynamic diameter at a 3.0 mg/mL concentration.

Figures 4 and 5 show the results of morphometric analyses of AM, NM and TM. Exposure to PM_2.5_ induced more structural changes in the frog epithelium than TSP. Urban particulate matter (PM_2.5_), even at the lowest dose, induced significant reductions of stored epithelial mucus substances in comparison to the control (*p*<0.05), suggesting that mucus secretor epithelial cells extruded their contents after exposure to traffic particles. In contrast, exposure to TSP determined the same behavior as the control group (Figure 4). PM_2.5_ exposure also induced a decrease in the epithelium thickness (*p*=0.01), as depicted in Figure 5, while TSP and AR solution did not induce any change.

## DISCUSSION

Our results showed that particles from vehicular sources and from a steel plant at real ambient concentrations acutely affected mucociliary function in different ways. Although both affected MCT, the traffic derived particles seemed more aggressive by decreasing the epithelial structural characteristics, apparently inducing shrinkage of the frog epithelium. These findings support the concept that the composition modulates the effects of real ambient particles on the mucociliary epithelium, likely through different physiopathological pathways. Further, these results agree with previous studies, indicating that the mucociliary system is a sensitive target for airborne particles 15-17.

As a test system, we used an isolated frog palate preparation, which presents a surface similar to the human respiratory epithelium and has been successfully used in toxicological studies 18. The indicators of effect were MCT, CBF and the amounts of acidic and neutral epithelial mucus substances. This set of indicators comprises both functional and structural abnormalities of the mucociliary apparatus.

Different particles were chosen for this study due to their differences in chemical composition and because they represent typical real sources: a stationary industrial source, in the case of TSP from sintering at a steel plant and traffic-generated particles from a large urban area of São Paulo city in the case of PM_2.5_. As expected, those particles emitted by the steel plant had greater concentrations of iron (Table 1) and negligible amounts of organic substances. In contrast, traffic-derived particles had greater amounts of sulfur, zinc, copper, nickel, vanadium and several organic species. These different chemical signatures elicited almost the same effects in terms of mucociliary function, as expressed in terms of mucus transportation. However, the morphological changes; i.e., decreases in stored mucus substances and, consequently, epithelial thickness, were more intense in the case of traffic particles. This finding indicated that sulfur compounds, metals and organics could play relevant roles in the epithelial structural injury elicited by urban aerosol.

The aggressiveness of the urban air of big cities in South America was shown by Bell et al. 19. In São Paulo city, they estimated that if pollutants decreased 10% from 2000 to 2020, it would reduce 114,000 deaths, 138,000 child and teenager visits to the hospital, 103,000 visits to emergency rooms due to respiratory diseases, 817,000 asthma attacks, 50,000 acute and chronic bronchitis events and 2.5 million days of absenteeism. In São Paulo state, public opinion in the last 2 decades has developed to desire control of air pollution emissions 20. Vormittag et al. 12 showed that those feasible public attitudes affected health indexes during 2006 to 2011 but also noted that they were not enough. Ambient PM_2.5_ was estimated as 22.17 µgm^-3^ in São Paulo state, including industrial and urban areas. São Paulo city has no industries because they have been reallocated to the countryside of the state. Areas with and without industries and rural zones with similar levels of particles (annual and 24-h mean values) had different proportions of respiratory diseases in adults and children. Respiratory events in adults and in children were 28% and 33% in São Paulo city, 41% and 19% in an industrial area and 33 and 31% in a rural zone, respectively. The DALY tax (per 1,000 inhabitants) and loss of days of life/year/habitant index estimations to premature death or disability due to pollutants in São Paulo city and SP state were 3.6, 3.8, 1.31 and 1.39, respectively. These data are in accordance with our study, suggesting that similar levels of particles from different sources affect the respiratory system differently and that the particle compositions are a key point in respiratory health effects.

The toxicity of metals contained in ambient particles has been demonstrated in several toxicological studies focusing on the respiratory system using either *in vivo* or *in vitro* approaches 21-26. Several metals have been suggested as potential agents in the biological response to PM, including iron, copper, vanadium, zinc and nickel. Several studies have suggested oxidative stress as the possible mechanism of toxicity for these first-row transition metals 6-7. Indeed, a study conducted by Ghio AJ and Devlin RB 28 demonstrated that ambient particles collected during the interruption of the activities of a steel mill in Utah were significantly less toxic when modulated by oxidative stress in comparison to those sampled during plant operations. Additionally, Macchione et al., 1999 16 showed that oxidative stress alters the function and structural properties of the mucociliary system.

Interestingly, most of the available literature on the effects of organics on the respiratory epithelium focuses on mutagenesis or carcinogenesis 29-31 because of the well-established relationship between organics, which are mostly polycyclic aromatic hydrocarbons and cancer 32-33. Further, Carvalho-Oliveira et al. 34 studied ambient particles during a one-day strike of bus drivers (stoppage of diesel vehicles) compared to an ordinary day (petrol and diesel vehicles) in São Paulo, Brazil. In this study, the authors demonstrated that, although the PM masses were the same on both days, their components were different (50% fewer organic compounds and sulfur), suggesting that diesel particles, which are mainly organics and sulfur compounds, induced more deleterious effects than those from petrol. However, studies focusing on acute toxicity of the organic fraction of ambient particles are less frequent. Both petrol and diesel fuels from vehicle combustion give rise to airborne PM. These particles contain a variety of organics in their structures, either adsorbed or trapped within the carbonaceous core. The complexity of the system is high, as transitions from the particle to the gas phase (and vice versa) occur depending on weather conditions 35. In addition, cross reactivity among particle constituents and photochemical processes may modify particle composition and, as a result, its toxicity. These chemical alterations, when particles are in flight in the urban air, are known as particle aging, an event that has biological consequences that have not been hitherto defined 36. Organic compounds adsorbed on PM can undergo complex cyclical chemical reactions in the milieu of the lungs that lead to the production of free radicals, such as superoxide anion or hydroxyl radical 37-38. The mucociliary system is extremely sensitive to oxidative stress, with the effects being detected at levels of oxidant load near sub-inflammatory conditions 16.

Although the results obtained in this acute investigation are in agreement with many experimental and epidemiological studies showing that air pollution particles injure the airway epithelium 15-16,, the extrapolation of these experimental data to human exposition in real ambient conditions should be taken with caution. First, evaluation was made in an *ex vivo* model, which cannot be directly correlated to the human epithelium. However, the pseudo-stratified epithelium of the frog palate is similar to that of mammals, with numerous ciliated and secretory cells with similar physical and chemical characteristics to those secreted by the human epithelium 18. Second, particles were diluted in AR because it is a well-established solvent to particles in the frog palate model 15-17. In the air, particles are suspended in the atmosphere and not diluted in an aqueous milieu. However, after inhalation, particles are diluted in the aqueous milieu of the airway epithelium. Third, the 3 mg/ml of PM_2.5_ concentration is much higher than the daily and annual means in the São Paulo atmosphere. We previously tested a PM_2.5_ dose response curve in the frog palate model in a pilot study. The 1 mg/mL starting PM_2.5_ dose was based on a prior study using the same experimental model 15. PM_2.5_ doses above 3 mg/ml blocked the frog palate response, while doses under 0.1 mg/mL did not elicit any effect on frog palates. Therefore, doses within this range were chosen for this study, focusing on acute responses to particles. Real-world traffic corridor exposure has much higher particle concentrations than daily and annual means and this type of exposure induces acute effects on health that are not easily measured and established. Therefore, although there is a gap between experimental controlled studies focusing on mechanistic pathways and real ambient exposure, this study contributed to the physiopathologic knowledge of particle effects on health.

Despite these limitations, the demonstration that different element compositions of the particulate matter produce different airway epithelial responses reinforces the idea that the potential of particles to cause injury varies with particle size and other physical characteristics, chemical composition and source(s). At this time, if different real ambient chemical compositions of PM induce different health effects, knowledge of the individual characteristics of PM may help to establish policies aimed at air pollution control.

## Figures and Tables

**Table 1:** Trace elements detected in particulate matter in TSP and urban PM_2.5_ samples determined by energy dispersive X-ray fluorescence. Values are expressed in ng/cm^2^.

Elements	PM2.5				TSP			
		Mean	SD	Min	Max	Mean	SD	Min	Max
**Al**	2092.87	3173.2	50	7560	243.3	86.79	163.71	381.24
**Ca**	6319.15	2056.4	2122.65	7246.96	17326.07	1029.77	16490.66	18746.2
**Cu**	295.68	159.75	201.45	619.71	47.14	11.23	30.61	60.01
**Fe**	5508.02	7114.9	741.4	19674.99	82507.29	6371.3	77378.4	91157.24
**K**	3447.3	4067.65	86.13	8965.63	12160.76	851.3	11182.87	13219.38
**Na**	6983.34	443.21	6442.02	7422	1646.66	253.39	1273.23	1996.43
**Ni**	118.85	90.7	2.24	225.81	ND	ND	ND	ND
**Pb**	58.75	88.4	0	221.52	1590.87	146.16	1483.27	1794.6
**S**	8148.23	4909.5	4005.02	16652.87	7666.33	474.09	7292.37	8293.68
**Si**	4197.89	4757.07	1.61	13223.91	5922.42	308.92	5574.73	6288.79
**Ti**	68.59	66.14	14.43	179.04	181.05	24.7	157.02	210.43
**V**	121.51	76.9	43.67	226.43	ND	ND	ND	ND
**Zn**	1208.02	767.27	80.81	2040.82	111.67	11.69	90	120
mass*	0.373	0.206	0.161	0.714	0.502	–	–	–

PM_2.5_: urban particulate matter (2.5 μm in aerodynamic diameter), TSP: total suspension particles collected from an electrostatic precipitator of a steel plant, ND: not detected, *: mass expressed in g.

**Appendix 1:** Values obtained for reference material NIST 2387 determined by energy dispersive X-ray fluorescence. Values expressed in ng/cm^2^.

	Certified values		Determined values	
Elements	Mean	SD	Mean	SD	RE
**Al**	2330.32	53.21	2004.8	16.36	-13.97
**Ca**	1325.3	170.7	1104.3	13.97	-16.67
**Cu**	40.56	4.22	38.74	1.55	-4.5
**Fe**	2660.64	160.6	3413.7	176.42	28.3
**K**	530.12	52.21	434.78	8.72	-17.99
**Na**	186.75	10.04	119.38	10.56	-36.07
**Ni**	6.83	1.2	6.18	1.15	-9.44
**Pb**	31.83	5.42	22.67	7.58	-28.78
**S**	105.42	26.1	87.77	0.93	-16.75
**Si**	5883.53	160.6	4956.7	12.41	-15.75
**Ti**	149.6	24.1	128.12	52.11	-14.36
**V**	4.87	0.6	4.5	1.6	-7.63
**Zn**	179.72	13.05	155.63	3.08	-13.4

SD, Standard Deviation; RE, Relative, Error
